# Making Ozonolysis Mass Spectrometry More Accessible: A Simple LC-OzESI-MS Workflow for Carbon–Carbon Double Bond Position Elucidation in Lipids and Natural Products

**DOI:** 10.1021/jasms.6c00205

**Published:** 2026-07-25

**Authors:** Fayaj A. Mulani, Ashraf M. Omar, Qibin Zhang

**Affiliations:** † Center for Translational Biomedical Research, 14616 University of North Carolina at Greensboro, North Carolina Research Campus, Kannapolis, North Carolina 28081, United States; ‡ Department of Chemistry & Biochemistry, University of North Carolina at Greensboro, Greensboro, North Carolina 27402, United States

## Abstract

Elucidation of C=C unsaturation sites in biomolecules remains challenging using conventional tandem mass spectrometry techniques. Online ozonolysis inside a mass spectrometer offers a very specific C=C fragmentation strategy; however, adoption of the precursor-selective ozone-induced dissociation has been limited by its low reaction yields and the need for specialized mass spectrometers that can provide higher partial pressure of ozone and/or modifications to enable effective ion trapping. To make the online ozonolysis mass spectrometry more accessible, we present a setup in which ozone is introduced directly into the sheath gas line of a standard heated electrospray ionization source. This minimally modified online ozonolysis setup enables efficient interaction of ozone with electrospray droplets at atmospheric pressure while maintaining normal instrument operation and safety. In combination with reversed-phase liquid chromatography separation, this setup is very effective in annotating C=C positions in unsaturated biomolecules with just MS1 scanning by relying on the coelution of precursors and the ozonolysis product ions. This approach was validated across multiple lipid classes—including fatty acids, glycerophospholipids, sphingolipids, glycerolipids, and cholesteryl esters—as well as selected natural products, in complex mixtures and under both positive and negative ion modes. Reaction yields varied among analytes yet remained sufficient for unambiguous C=C position assignment. This LC-OzESI-MS strategy provides an efficient and easily adoptable platform for high-throughput structural characterization of unsaturated biomolecules, even in complex sample matrices.

## Graphical abstract

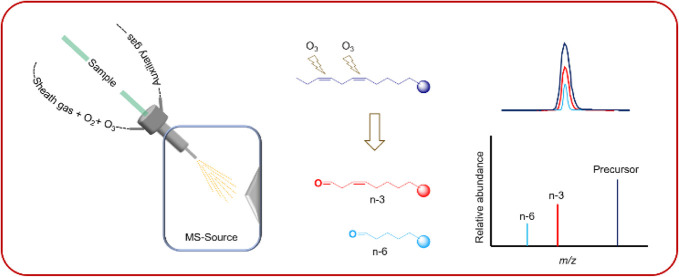

## Introduction

The function of a biomolecule is determined by its structure. In particular, variations in C=C double bond placement are known to affect signal transduction and metabolism and have been connected to several diseases.^1–4^ Full structural elucidation needs the extensive use of different spectroscopic techniques, such as nuclear magnetic resonance (NMR) and mass spectrometry (MS). The latter has been at the center focus of the research in this field due to its ability, in most cases, to pinpoint the exact mass, elemental composition, and structural subunits of the molecule under investigation. However, when it comes to molecules carrying fully substituted double bonds or multiple double bonds in similar chemical environments, such as aliphatic side chain(s), NMR and conventional tandem mass spectrometry (MS/MS) techniques are often insufficient in pinpointing the exact position(s) of C=C and typically provide information only at the level of the total degree of unsaturation.^5^ Therefore, developing MS-based methodologies capable of deciphering C=C regioisomerism has been the focus of a multitude of research for the past decade.

Many technologies have emerged for that purpose, including direct analysis methods using mass spectrometers equipped with specialized ion dissociation techniques such as ultraviolet photodissociation^6,7^ and radical-induced dissociation^8^ as well as in-solution chemical derivatization of molecules prior to MS analysis. These reactions include epoxidation,^9,10^ aziridination,^11–14^ Paternò-Büchi reaction,^15–18^ and ozonolysis.^19^ The latter has been extensively studied due to the simplicity of the spectrum generated. Its unique chemistry arises from the reaction of ozone with C=C through a Criegee-type mechanism, whereby the double bond undergoes electrophilic addition to form a primary ozonide. This unstable intermediate rapidly decomposes to yield C=C position-characteristic aldehyde and Criegee species (Figure 1A). The exact mass differences between these diagnostic fragments and the precursors enable unambiguous localization of double-bond positions within fatty acyl chains or other unsaturated structures. Traditionally, ozonolysis was performed in-solution, followed by chromatographic and mass spectrometric analysis of the resulting products.^20^ While effective, the requirement of offline derivatization and subsequent analysis limiting their utility for high-throughput studies.

**Figure 1:**
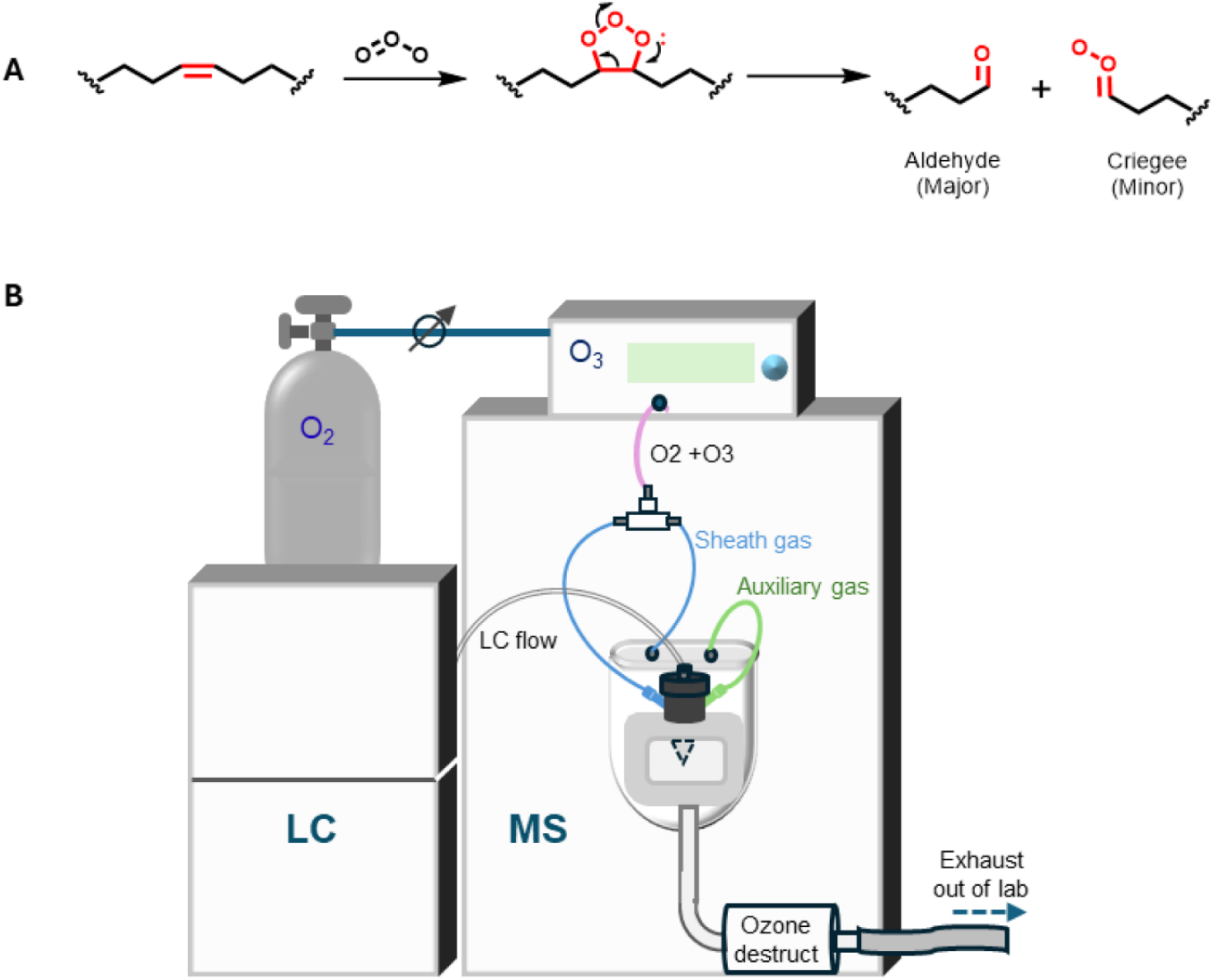
OzESI reaction mechanism and instrument setup. A) OzESI ion chemistry. Unsaturated lipids react with O_3_ to form the C=C position-specific aldehydes and Criegee fragments. B) Modifying the HESI source by adding O_2_ and O_3_ to the nitrogen sheath gas using a T-junction.

In situ coupling of ozonolysis to MS opened a new path by translating ozonolysis from in-solution to the gas phase. Ozone-induced dissociation (OzID) is the most well-known online ozonolysis technique, in which ozone is plumbed into the collision cell of a mass spectrometer, the mass-selected precursor ions react with ozone gas to produce C=C position-specific aldehyde (major) and Criegee (minor) ions.^21,22^ Because of mass selection, OzID is very specific, but the sensitivity (yield) of this first-order reaction is typically very low, given the low partial pressure of ozone (a few millitorr) inside the collision cell. To improve yield, OzID has been implemented in the ion mobility cell^23^ and high-pressure ion funnel,^24^ where the gas pressure can reach a few torr, or by using ion mobility-enabled ion trapping to prolong the reaction time;^25^ however, all of these require specialized mass spectrometers or modifications to the instrument control software. An earlier developed, yet less-known online ozonolysis technique, termed OzESI,^26^ unlike OzID where ozonolysis occurs after ionization, the ozonolysis in OzESI occurs concurrently with the electrospray process. Ozone in OzESI is either generated in the corona discharge in the electrospray ionization process^19^ or generated exogenously from an ozone generator and plumbed into the nebulizing gas of the ionization source.^26^ Due to its operation at atmospheric pressure and potentially accelerated reactions in microdroplets,^27^ OzESI has a much higher yield in the production of C=C specific aldehyde ions, but it lacks specificity, especially when used in direct infusion MS settings. Coupling with liquid chromatography (LC) separation, we have used LC-OzESI-MS for structural annotation of C=Cs in natural products,^28,29^ and most recently, Randolph et al. developed an LC-OzESI-MRM workflow for comprehensive structural elucidation of triacylglycerols,^30^ and Xu et al. established a comprehensive workflow for lipidome-level C=C determination, incorporating ozonolysis at the ionization source.^31^ All of these rely on the coelution of the precursor and C=C specific aldehyde/Criegee ions in the MS1 scan for confident unsaturation site assignment.

In this study, we present a comprehensive evaluation of LC-OzESI-MS in C=C position determination with various classes of lipids, including fatty acids, phospholipids, glycerolipids, ceramides, and natural products. We show that when coupled with reversed-phase (RP) LC separation, OzESI at the MS1 level is quite capable of annotating C=C positions in lipids, even in relatively complex mixtures and lipid extracts from biological samples. All work was performed on a Thermo mass spectrometer with a heated electrospray ionization (HESI) source with minimal instrument modification.

## Methods

### Materials

Lipidomics standard mixture (UltimateSPLASH ONE, Catalog #: 330820) and Mouse SPLASH Standard (Catalog #: 330710) were purchased from Avanti Polar Lipids (Alabaster, AL, USA). Standards of fatty acids were purchased from Cayman Chemical (Ann Arbor, MI, USA), CDN isotopes (Ontario, Canada), and NU-CHEK (Elysian, MN, USA). Cardol diene and coenzyme Q10 standards were acquired from Cayman Chemical. Solvents and reagents were acquired from ThermoFisher Scientific (Atlanta, GA). The fatty acid derivatization kit (AMP+ MaxSpec Kit) was purchased from Cayman Chemical and used according to the manufacturer’s protocol.

### Lipid Extraction

Lipids were extracted from a Non-Obese Diabetic mouse liver (collected under IACUC approval #23-002) homogenate using the Folch method. In brief, 40 μL of homogenate, equivalent to 2 mg of wet tissue, was mixed with 700 μL of CHCl_3_/MeOH (2:1), containing 5 μL of UltimateSPLASH, in a 1.5 mL Eppendorf tube. The tube was then vortexed for 5–10 s, followed by shaking on an Eppendorf thermomixer at 900 rpm for 1 h at 4 °C. This was followed by the addition of 200 μL of water, inducing the phase separation of two liquid phases. The tubes were vigorously vortexed for 10 s and centrifuged at 17,500 × *g*, 4 °C for 5 min, followed by the collection of the lower chloroform phase, then dried under a nitrogen stream and reconstituted in 60 μL of ACN/IPA/H_2_O (65:30:5, v/v/v).

### LC-MS Conditions

All LC-MS analyses were carried out using a Vanquish Horizon UHPLC (ThermoFisher Scientific) coupled to a Q Exactive HF mass spectrometer (ThermoFisher Scientific). The separation was performed on an ACQUITY Premier CSH C18 column (100 × 2.1 mm, 1.7 μm, Waters). Mobile phase composition, LC gradient conditions, and MS acquisition parameters were reported previously^32^ and have been modified in the current work. In brief, mobile phase A consisted of ACN:H_2_O (1:1, v/v), and mobile phase B consisted of IPA:ACN:H_2_O (88:10:2, v/v/v). Both mobile phases contained 5 mM ammonium formate and 0.1% formic acid, except the formic acid was omitted in the mobile phases in separation of free fatty acids. A flow rate of 230 μL/min was used, with the column temperature maintained at 52 °C. The LC gradient was as follows: 0.0–1.0 min (10% B), 2.0 min (30% B), 3.5 min (50% B), 7.0 min (60% B), 17.0 min (70% B), 18.0 min (80% B), 20.0 min (95% B), 22.0–28.0 min (100% B), and 28.1–32.0 min (10% B). Prior to each injection, the LC column was equilibrated for 5 min at 10% solvent B.

For mass spectrometry parameters, the HESI source settings for ozone on were as follows: sheath gas (2 Arb), auxiliary gas (5 Arb), sweep gas (1 Arb), spray voltage (3 kV), capillary temperature (250 °C), s-lens RF level (50), and auxiliary gas heater temperature (200 °C). Full MS scans were acquired over an *m*/*z* range of 200–1700 at a resolution of 120,000, with an AGC target of 1e6, and a maximum injection time of 250 ms in both positive and negative ion modes. For the ozone-off data-dependent MS/MS runs, the settings were: resolution of 30,000, AGC target of 1e5, maximum injection time of 75 ms, loop count of 10, isolation window of 1.0 *m*/*z*, stepped NCE of 25 and 30, and dynamic exclusion of 8 s.

### OzESI Setup

Very minor modifications to the HESI ion source were made to allow OzESI analysis. Briefly, a Titan-30 ozone generator (Absolute Ozone, Edmonton, Canada) was operated at an oxygen gas flow of 0.2 L/min at 20 PSI and power-adjusted to have ∼10% ozone output, as measured by an in-line 106-H ozone monitor (2B Technologies, Broomfield, CO, USA). Ozone was introduced via a Teflon tubing (1/16″ ID x 1/8″ OD) to the nitrogen sheath gas line by a PEEK T-junction (Figure 1B). The residual ozone was passed through an ozone destruct unit (Ozone Solutions, Hull, IA, USA) and vented to the laboratory exhaust under light vacuum. The entire setup was monitored for ozone leaks with a 106-L ozone monitor (2B Technologies), and the ambient ozone concentration was below 0.1 ppm throughout the experiment.

### Data Analysis

FreeStyle (V1.8, ThermoFisher) was used to process all acquired LC-MS data for extracted ion chromatograms. Annotation of C=C position is based on the signature neutral losses reported by Blanksby and coworkers.^2^ Throughout this work, the C=C position is labeled as n-#, where # is counted from the methyl end of the alkyl chain, and for relative comparison of the ozonolysis efficiency, the yield is defined as the ratio between the intensity of the aldehyde ion and of the remaining precursor ion in the spectrum. In addition, for the analysis of complex lipids extracted from mouse liver tissue, MS-DIAL (version 5.5.250627) was used to identify the unsaturated lipids from the ozone-off LC-MS/MS data file, and then its *m*/*z* and retention time were used as the basis in FreeStyle for targeted analysis of the C=C position in the ozone-on LC-OzESI-MS data file.

## Results and Discussion

### The New OzESI Setup

The rationale behind this study is to apply a minimal modification to the MS instrument, which would allow laboratories having Thermo MS with HESI ion sources to adopt these modifications without much technical challenge. This was achieved by introducing inline-generated ozone gas into the sheath gas flow path of the HESI source. As shown in Figure 1B, ozone was generated through an ozone generator using oxygen as the feed gas at 20 PSI and a flow rate of 0.2 L/min. The ozone/oxygen mixture output from the ozone generator was connected through a PEEK T-junction to the nitrogen sheath gas outlet of the mass spectrometer. The output of the T-junction was then connected to the sheath gas inlet on the HESI probe. The ozone generator produced ∼10% ozone in oxygen, which is sufficiently concentrated to produce the characteristic aldehyde ions in high yield during in-source ionization/ozonolysis reactions, making it ideally coupled with LC-MS workflows for the analysis of lipids and natural products. Any potential ozone leakage was monitored by an ozone monitor and the atmospheric ozone concentration near the instrument was <35 ppb, below the 0.1 ppm exposure limit permitted by the Occupational Safety and Health Administration (OSHA). Multiple factors contributed to the absence of hazardous leakage of ozone: 1) HESI source operates at ∼250 °C, and ozone decomposes into oxygen when exposed to heat; 2) the HESI source was vented to a lab exhaust system operated under light vacuum; and 3) an ozone destruct unit was installed on the exhaust line, which also converts ozone to oxygen. It is worth noting that this instrument modification does not affect normal MS analyses that do not need ozone. In that case, a simple switch off of the oxygen/ozone flow meter can restore the sheath gas flow directly to the HESI source without the need to disassemble the T-junction.

### LC-OzESI-MS Analysis of Fatty Acids

Free fatty acids are typically analyzed in negative electrospray ionization mode, although the ionization efficiency is limited for those ones with very long chains.^33^ Figure 2A shows the LC-OzESI-MS chromatograms of a fatty acid standard mixture containing 24 unsaturated fatty acids, extracted for the precursor [M-H]^−^ ion and the corresponding C=C position-specific aldehyde ions resulting from online ozonolysis, all in MS1 scans. For each unsaturated fatty acid, clearly the precursor and the corresponding aldehyde ions coelute. Of note that the unambiguous assignment of the C=C position is owing to this coelution relationship, which makes the aldehyde ions stand out in a generally not-clean MS1 spectrum at mass ranges (*m*/*z* < 400) of fatty acids. Under our chromatographic conditions, all 24 fatty acids have baseline separation. The elution order of the fatty acids with the same degree of unsaturation follows the increasing order of chain length, i.e., FA 12:1 < FA 15:1 < FA 16:1 < FA 17:1 < FA 18:1 < FA 19:1 < FA 20:1 < FA 21:1 < FA 22:1 < FA 24:1, while the elution order of fatty acids with the same chain length but different degrees of unsaturation is reversed, i.e., a higher degree of unsaturation elutes earlier, as in FA 22:6 < FA 22:5 < FA 22:4 < FA 22:3 < FA 22:2 < FA 22:1, all agreeing with the retention behavior of lipids expected on a reversed-phase LC column. The yield of aldehyde ions varied depending on the C=C position and the degree of unsaturation but can reach 60% or more in comparison to the remaining precursor ions. For example, all 6 aldehyde ions of FA 22:6 were observed (Figure 2C), at *m*/*z* 301.1806 (43%), 261.1493 (54%), 221.1177 (62%), 181.0862 (62%), 141.0546 (63%), and 101.0231 (2%), arising from a neutral loss (NL) of 26, 66, 106, 146, 186, and 226, which corresponds to a C=C present at *n*-3, *n*-6, *n*-9, *n*-12, *n*-15, *n*-18, positions of the 22:6 fatty acid.

**Figure 2:**
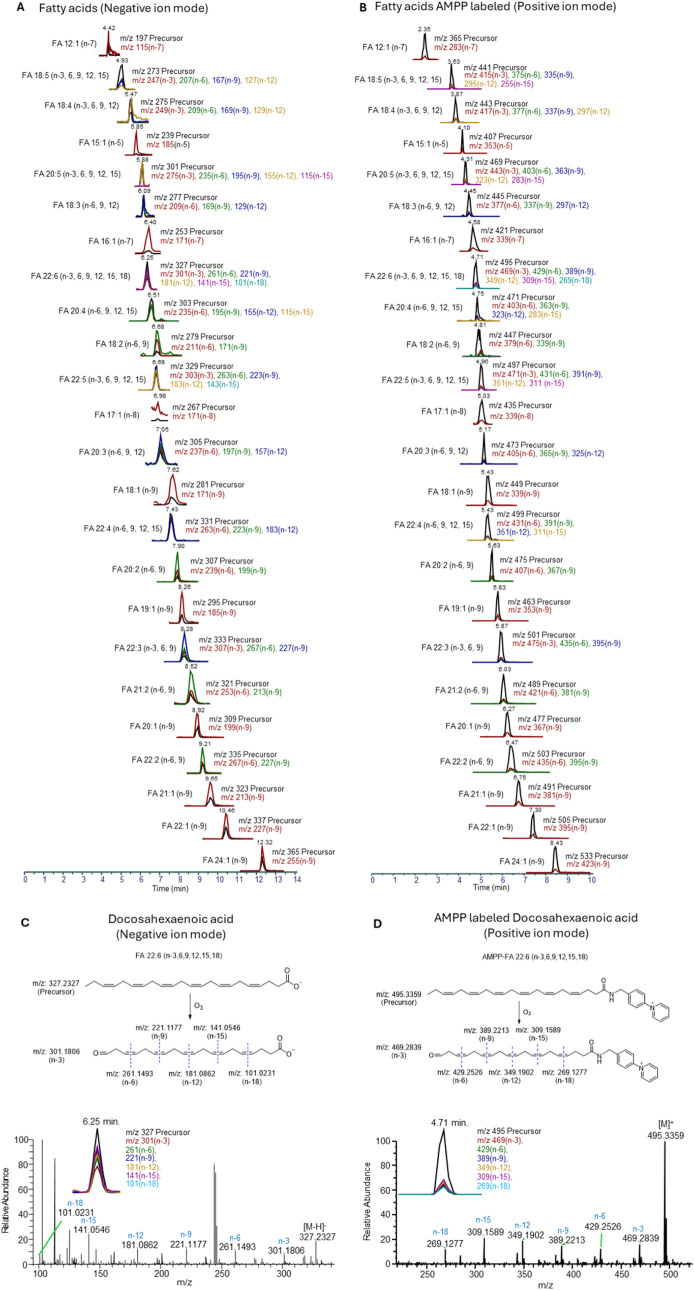
Selected ion chromatograms of fatty acids and representative mass spectra. A) Unlabeled fatty acids and B) AMPP-labeled fatty acids for the precursors and their respective C=C position-specific aldehyde ions. Representative mass spectra of C) unlabeled and D) AMPP-labeled fatty acid FA 22:6.

Fatty acids are also commonly chemically derivatized before their analysis using LC-ESI-MS. In this respect, charge reversal derivatization with 1-(4-(aminomethyl)­phenyl)­pyridinium (AMPP) has long been used to improve the analytical sensitivity of fatty acids. We explored LC-OzESI-MS analysis of the AMPP-derivatized fatty acids in positive ionization mode. The results showed that baseline separation was still achieved with these 24 unsaturated fatty acids, with the elution order following the same pattern as observed for the free fatty acids (Figure 2B), despite the fact that the fixed positively charged AMPP residue makes the elution of every derivatized fatty acid faster compared to the underivatized ones. This also allowed the enhanced selectivity of fatty acid detection by MS compared to the analysis in negative mode,^1,34,35^ as in the case of FA 17:1 (n-8), where high background interference to the aldehyde ion *m*/*z* 171 (Figure 2A) was observed in the analysis of free fatty acid but no interference to the AMPP-aldehyde ion *m*/*z* 339 as evidenced by the flat baseline of this ion (Figure 2B). Just like the negative mode LC-OzESI-MS of free fatty acids, perfect coelution was also observed in positive mode for LC-OzESI-MS of AMPP-derivatized fatty acids. In general, the yield of aldehyde ions was lower in positive mode for AMPP-derivatized fatty acids in comparison to the free fatty acids in negative OzESI mode, for example, the 6 aldehyde ions of AMPP-FA 22:6 were observed at *m*/*z* 469.2839 (15%) for n-3, 429.2526 (11%) for n-6, 389.2213 (13%) for n-9, 349.1902 (17%) for n-12, 309.1589 (21%) for n-15, and 269.1277 (12%) for n-18, respectively (Figure 2D). Despite the yield being lower, the abundance is still high enough for unambiguous assignment of the C=C location, with an additional benefit of a cleaner spectrum, likely due to the upper shift of the mass range because of AMPP addition.

### LC-OzESI-MS Analysis of Glycerophospholipids

We next applied this LC-OzESI-MS setup for the analysis of unsaturated glycerophospholipids, in both ionization modes, by analyzing ultimateSPLASH standards, a mixture containing 69 unique lipids across 15 lipid classes, including 53 unsaturated lipids. Again, all C=C-specific aldehyde ions resulted from online ozonolysis coeluted with their respective unsaturated lipid precursor ions (Figures 3 and 4). For phosphatidylinositol (PI), the yield of aldehyde ions for PI-*d*_5_ 17:0/16:1 (*n*-7) was 17% and 15% relative to the precursor ions, respectively, under negative and positive ionization modes (Figure 3). Lipids belonging to the phosphatidylserine class (PS) were similar to the PI species. The PS-*d*_5_ 17:0/20:3 (*n*-6,9,12) gave the corresponding OzESI products at *m*/*z* 735.4604 (14%), 695.4293 (13%), and 655.3976 (12%) under negative ion mode and 737.4734 (12%), 697.4423 (13%), and 657.4113 (16%) under positive ion mode, formed at C=C positions of *n*-6, *n*-9, and *n*-12, respectively. Another tested lipid class is phosphatidylglycerol (PG). The OzESI analysis of PG-*d*_5_ 17:0/22:4 (*n*-6,9,12,15) revealed the formation of OzESI products at *m*/*z* 748.4806 (18%), 708.4494 (20%), 668.4182 (27%), and 628.3865 (25%) in negative ion mode and 767.5203 (12%), 727.4891 (11%), 687.4579 (12%), and 647.4267 (11%) in positive ion mode, corresponding to n-6, n-9, n-12, and n-15, respectively (Figure 3). It is of note that PI, PS, and PG classes of lipids all had precursors of [M-H]^−^ in negative mode, while [M + H]^+^ for PS, and [M+NH_4_]^+^ for PI and PG in positive ion mode.

**Figure 3:**
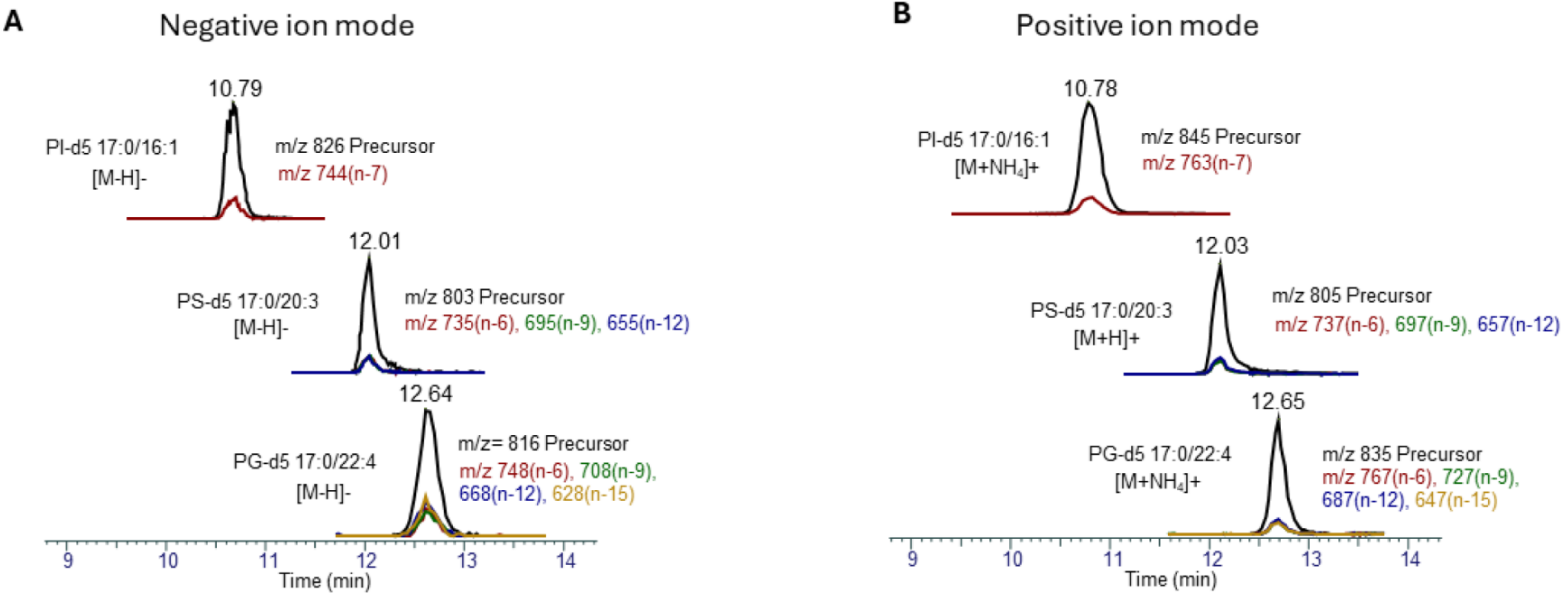
LC-OzESI-MS selected ion chromatograms of the precursors and their respective C=C position-specific aldehyde ions for PI (PI-d5 17:0/16:1), PS (PS-d5 17:0/20:3), and PG (PG-d5 17:0/22:4) lipid classes in A) negative and B) positive ion mode.

**Figure 4:**
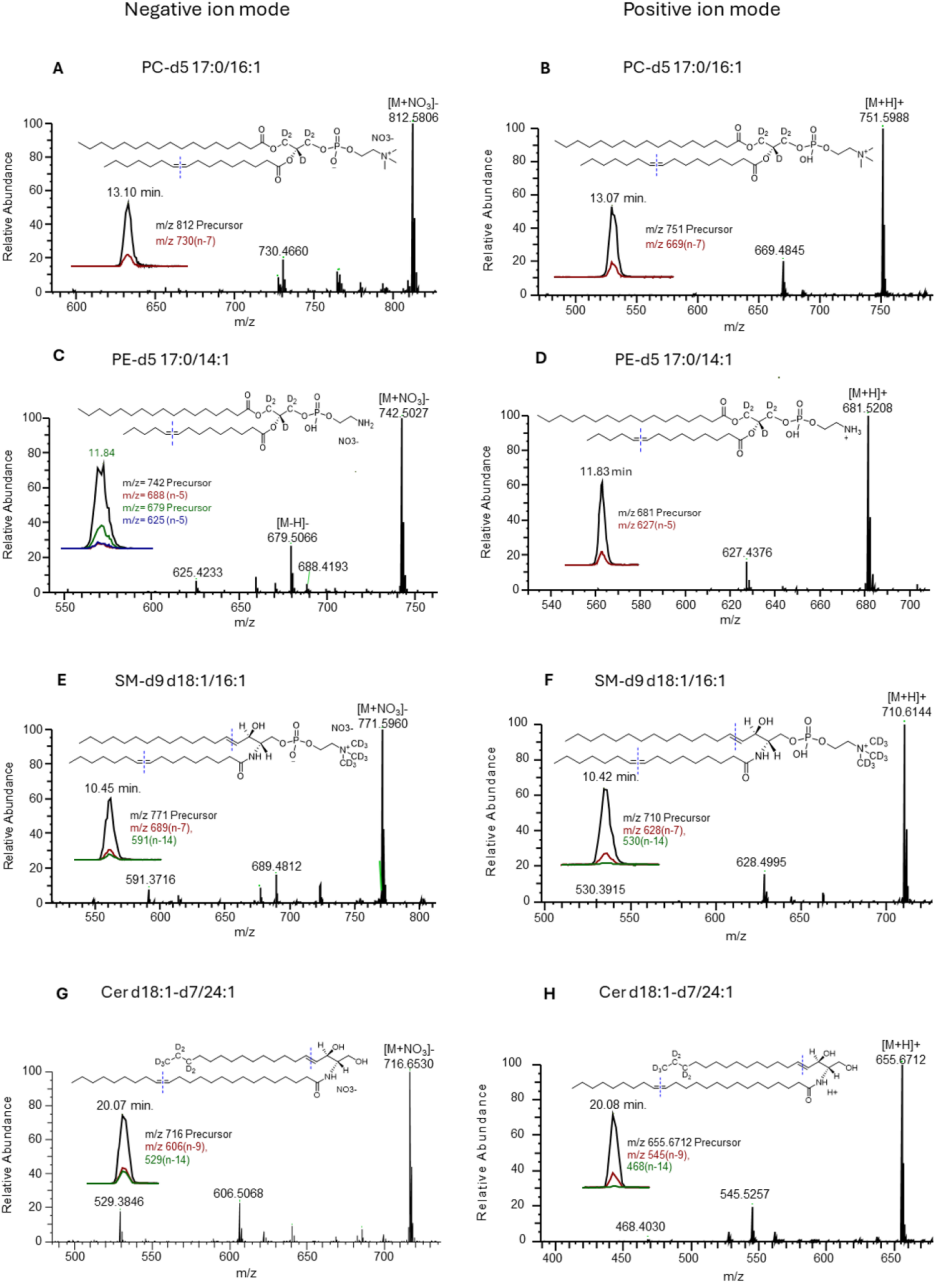
LC-OzESI-MS selected ion chromatograms and mass spectra of lipids from class PC, PE, SM, and Cer, both in negative ion mode and positive ion mode, respectively. (A, B) PC-d5 17:0/16:1; (C, D) PE-d5 17:0/14:1; (E, F) SM-d9 d18:1/16:1; (G, H) Cer d18:1-d7/24:1.

Under our experimental conditions in negative ion mode, phosphatidylcholine (PC) formed a novel, predominant [M+NO_3_]^−^ adduct as the precursor ion, which was not observed from the other aforementioned glycerophospholipid classes. For example, *m*/*z* 812.5806 was observed as the precursor of PC- d_5_ 17:0/16:1 (*n*-7), and the neutral loss of 82 gave rise to the aldehyde OzESI product at *m*/*z* 730.4660 (19%), corresponding to a C=C present at *n*-7 position of the 16:1 fatty acyl (Figure 4A). Similarly, for phosphatidylethanolamine (PE) PE-*d*_5_ 17:0/14:1 (*n*-5), *m*/*z* 742.5027 ion is the dominant adduct, much more intense than the *m*/*z* 679.5066 deprotonated adduct of the precursor (Figure 4C), the yield of aldehyde ions (*m*/*z* 688.4193 and 625.4233) from the [M+NO_3_]^−^ and [M-H]^−^ were observed at 5.2% and 23.3%, respectively to their precursors. Exact mass measurement and isotopic envelope pattern confirmed the adduct assignment of [M+NO_3_]^−^. We found that this adduct even exists, albeit at much lower intensity (∼1%), under regular ESI conditions with N_2_ as sheath gas, and its intensity is much higher when O_2_ and O_3_ are included in the sheath gas mixture, which is very likely due to the HNO_3_/NO_3_^–^ species resulted from dielectric barrier discharge of gas environments containing O_2_ and N_2_,^36^ as observed under atmospheric pressure matrix-assisted laser desorption ionization with plasma post-ionization in imaging of lipids.^37^ On the other hand, in positive ion mode, [M + H]^+^ was observed as the precursor ion for the PC and PE species, with an OzESI aldehyde product at *m*/*z* 669.4845 (19.3%) and *m*/*z* 627.4376 (16.0%) from the same neutral losses (Figure 4B and D) as in negative ion mode.

### LC-OzESI-MS Analysis of Sphingolipids

Sphingolipids are composed of a long-chain aliphatic amino alcohol, also called a sphingoid long-chain base, and an N-linked fatty acyl. Unsaturation could exist either in the long-chain sphingoid base subunit or in the N-linked fatty acyl. Two species were investigated in this study, representing two sphingolipid subclasses: sphingomyelins (SM) and ceramides (Cer). Similar to PC, in negative ion mode, SM-*d*_9_ d18:1­(*n*-14)/16:1­(*n*-7) formed an [M+NO_3_]^−^ adduct at *m*/*z* 771.5960, online ozonolysis resulted in the formation of the aldehyde products *m*/*z* 689.4812 (16%) and 591.3716 (8%), corresponding to the C=C on the fatty acyl (n-7) and the sphingoid base (n-14), respectively, with the fatty acyl-derived ion more abundant than the one from the sphingoid base (Figure 4 E). Also like PC, the [M + H]^+^ ion is dominant for SM in positive ion mode. Online ozonolysis produced aldehyde ions at *m*/*z* 628.4995 (15%) and 530.3915 (2%), corresponding to the respective fatty acyl and the sphingoid base (Figure 4F), again with the fatty acyl-derived aldehyde ion much more abundant than the one from the sphingoid base.

Reactivity of Cer-d_7_ d18:1­(*n*-14)/24:1­(*n*-9) was similar to SM, i.e., the formation of [M+NO_3_]^−^ adduct of the molecular ion in negative ion mode and the [M + H]^+^ in positive ion mode. Also, the reaction yield of ozone with fatty acyl was higher than that with the sphingoid base. In this regard, the relative intensity of the aldehyde ion from the n-9 fatty acyl at *m*/*z* 606.5068 was 22%, while that from the n-14 sphingoid base was 17% at *m*/*z* 529.3846 in negative ion mode. The difference was much larger in positive ion mode, with the relative intensity of the aldehyde ion from the n-9 fatty acyl at 19% (*m*/*z* 545.5257), while that from the n-14 sphingoid base was barely detectable at 1% (*m*/*z* 468.4030) (Figure 4 G and H).

The general observation of higher reactivity of C=C with ozone in fatty acyl, compared to that in the long-chain sphingoid base, is consistent with what reported in the literature,^38,39^ likely due to the gas-phase conformation of Cer and SM ions, which may limit the exposure of the sphingosine C=C to ozone. The differential reactivity indicates that, for the very low-abundance sphingolipids, the sphingoid C=C may not be reliably assigned, which may affect both the identification and semiquantitation of those unsaturated sphingolipids.

### LC-OzESI-MS Analysis of Glycerolipids and Cholesteryl Ester

We also tested this LC-OzESI-MS setup with two classes of unsaturated, hydrophobic lipids, triacylglyceride (TG), and cholesteryl ester (CE). Both classes of lipids only ionize well in positive ion mode with [M+NH_4_]^+^ as the major adduct. For the monounsaturated TG-*d*_5_ 16:0/15:1­(*n*-5)/16:0, precursor ion *m*/*z* 813.7686 gave rise to aldehyde ion *m*/*z* 759.6855, which was observed at 22% relative intensity, indicating n-5 placement of the C=C position (Figure 5A). The triunsaturated CE-*d*_7_ 20:3 (*n*-6,9,12), on the other hand, had three very abundant aldehyde ions at *m*/*z* 631.5775 (232%), 591.5462 (181%), and 551.5148 (128%) from precursor ion *m*/*z* 699.6755, for the n-6, n-9, and n-12 C=C, respectively (Figure 5B). Such a high yield of the OzESI product in cholesterol ester is likely the result of a favorable conformation exposing C=C to ozone.

**Figure 5:**
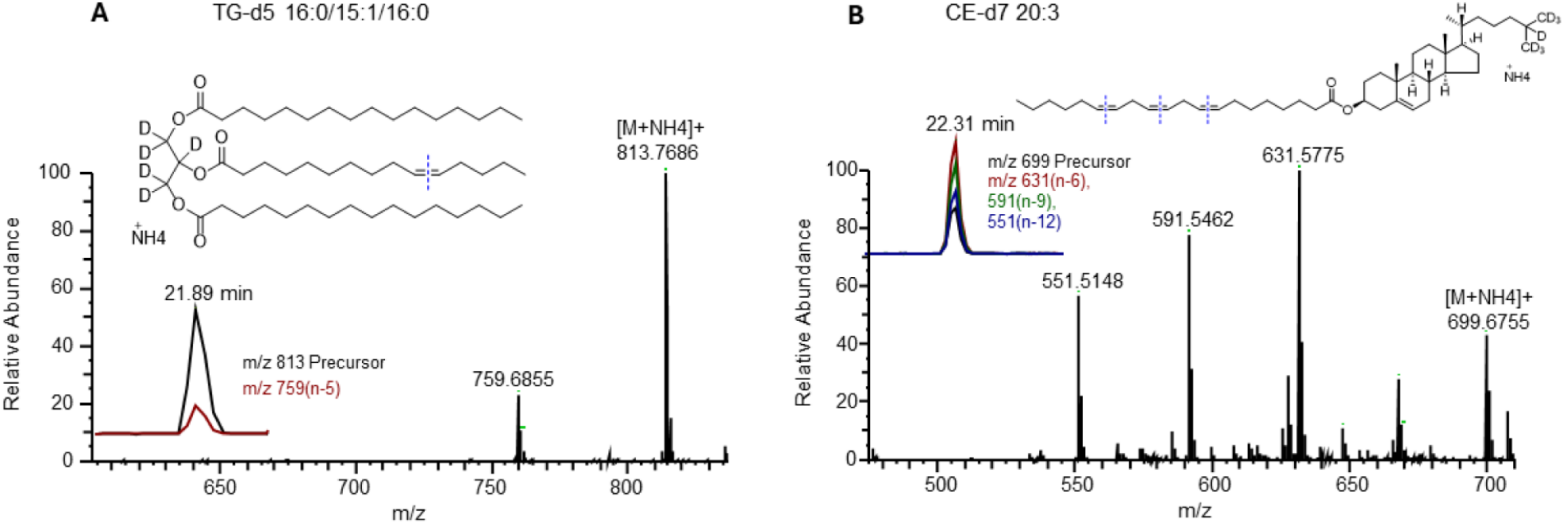
LC-OzESI-MS selected ion chromatograms and mass spectra of A) TG-d5 16:0/15:1/16:0 and B) CE-d7 20:3.

### LC-OzESI-MS Analysis of Selected Natural Products

Besides lipids, natural products, the secondary metabolites from plants, fungi, or other living organisms, contain C=Cs that can be challenging to assign their positions using traditional NMR techniques due to the tiny chemical shift differences of the C=Cs in long-chain hydrocarbons. We previously reported the application of direct infusion-based online ozonolysis in the trap region of a mass spectrometer for placing C=Cs in fungal metabolites containing conjugated or nonconjugated polyenes, where the C=Cs are either in the linear hydrocarbon or inside a ring structure.^5^ This was followed by us applying LC-OzESI-MS for C=C placement in two kinds of natural products, a cyclic lipodepsipeptide, Orfamide N, isolated from the *Pseudomonas idahonensis* bacterium^28^ and a natural long-chain monounsaturated aldehyde, (Z)-docos-15-enal, isolated from a sea sponge species.^29^

Here in this work, we further applied LC-OzESI-MS to two types of natural products that are isolated from plants: cardol diene and coenzyme Q10 (CoQ10). Cardol diene, isolated from cashew nut, is a potent antiparasitic agent with schistosomicidal activities against *Schistosoma mansoni* worms.^40^ Structurally, it is a 5-alkylresorcinol in which the resorcinol subunit is connected to a C15:2 alkyl moiety having two double bonds located at n-4 and n-7 (Figure 6A). The location of the two double bonds on the alkyl unit was confirmed through the specific aldehyde ions generated from the ozonolysis reaction at *m*/*z* 277.1791 (417%) and 237.1476 (99%), corresponding to the n-4 and n-7 C=Cs, respectively (Figure 6A) and the coelution of the aldehyde ions with the [M + H]^+^ precursor ion. Unlike the aliphatic C=Cs, no aldehyde products were detected corresponding to the benzene (phenolic) ring. CoQ10 is a ubiquinone containing 10 repeating isoprene units and is commonly used as a dietary supplement for heart health. Plant origins of CoQ10 include nuts and some vegetables. When subjecting CoQ10-d_6_ to LC-OzESI-MS analysis in positive ion mode, C=C-specific aldehyde ions at *m*/*z* 860.7034 (n-2, 155%), 792.6370 (n-6, 176%), 724.5756 (n-10, 292%), 656.5132 (n-14, 469%), 588.4509 (n-18, 804%), 520.3880 (n-22, 1368%), 452.3264 (n-26, 2373%), 384.2640 (n-30, 2344%), and 316.2014 (n-34, 1524%) were observed, all ions coeluting on the LC with the [M+NH_4_]^+^ ion of the precursor. Also coeluted are *m*/*z* 435.2997 (n-26, 1080%), 367.2376 (n-30, 3186%), 299.1751 (n-34, 2670%), and 231.1134 (n-38, 17%), except these four ions were detected as [M + H]^+^ (Figure 6B). Comparing the reactivity of ozone with C=Cs at different positions, the isoprenyl C=Cs at n-18, n-22, n-26, n-30, and n-34 of the aliphatic chain had very high reactivity, on the contrary, ozone reactivity of the C=Cs in the benzoquinone ring was much lower. The ultrahigh reactivity toward ozone may serve as a marker for the existence of isoprenyl units in unknown natural-product analysis.

**Figure 6:**
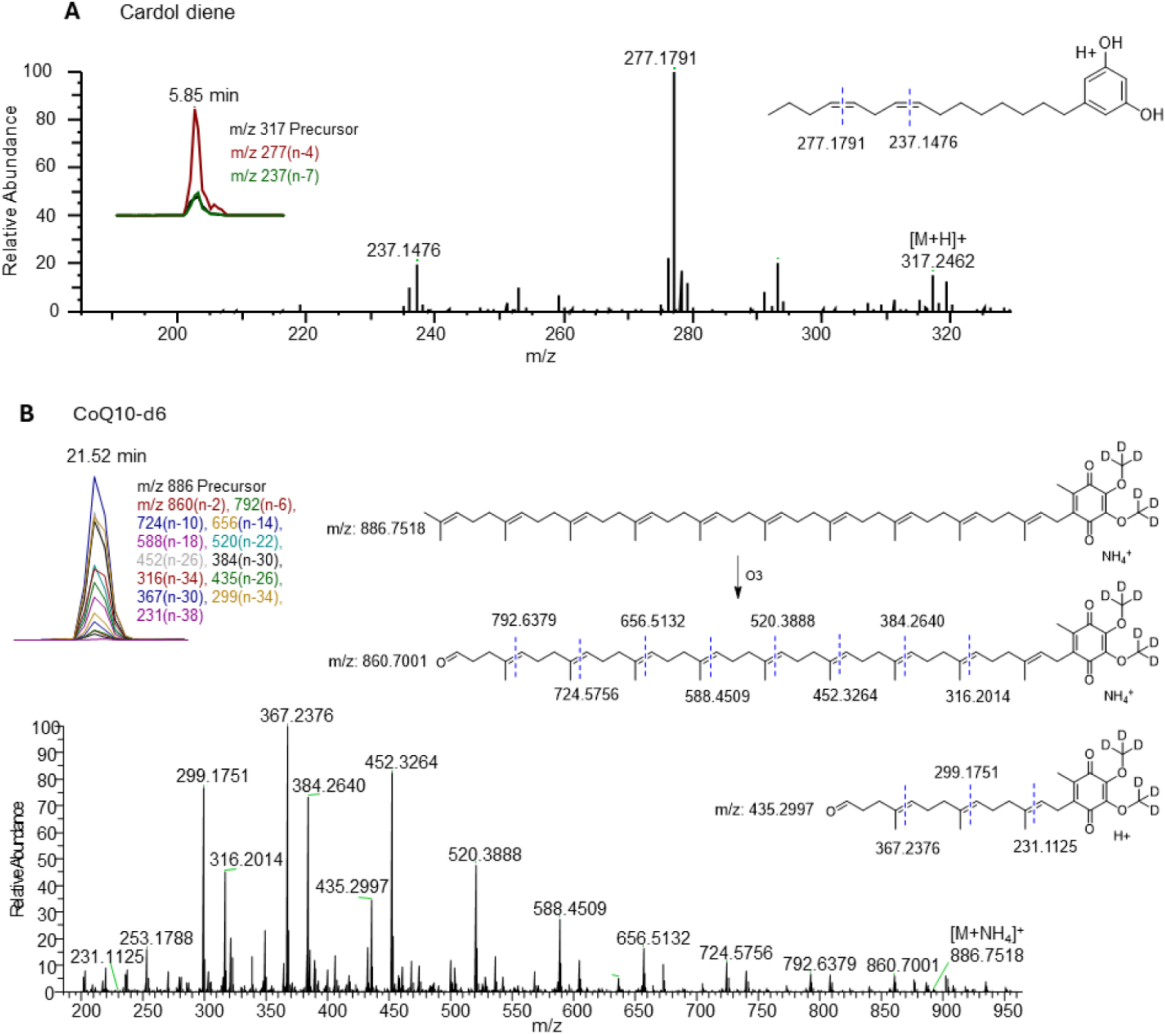
LC-OzESI-MS selected ion chromatograms and mass spectra of natural products A) Cardol diene and B) CoQ10-d6.

### LC-OzESI-MS Analysis of LysoPC *Sn*-Positional Isomers

The LC-OzESI-MS setup is also capable of distinguishing lipid *sn*-isomers. When analyzing the Mouse SPLASH standard mixture, which contains unsaturated lysophosphatidylcholines (LPCs) in positive ion mode, two peaks were observed at RT 5.20 and 5.36 min, both showing the same *m*/*z* 529.4006 for the [M + H]^+^ precursor ion of LPC 18:1-d7, the same OzESI aldehyde product was also observed at *m*/*z* 412.2102, indicating C=C position at the same n-9 position, but with different yields, 49% for the minor, early-eluting peak and 26% for the major, late-eluting peak (Figure 7). Since this LPC species was supplied commercially as an *sn*-1 isomer, then the major, late-eluting peak is the *sn*-1 isomer, while the early-eluting one is the *sn*-2 isomer. This agrees with the eluting order of *sn*-isomers on a reversed-phase LC column,^41^ the minor *sn*-transacylation occurred to *sn*-1 lipids, and the difference in yield between the *sn* isomers, for which it was reported that *sn*-2 isomer of PC has a higher ozonolysis yield.^25^

**Figure 7:**
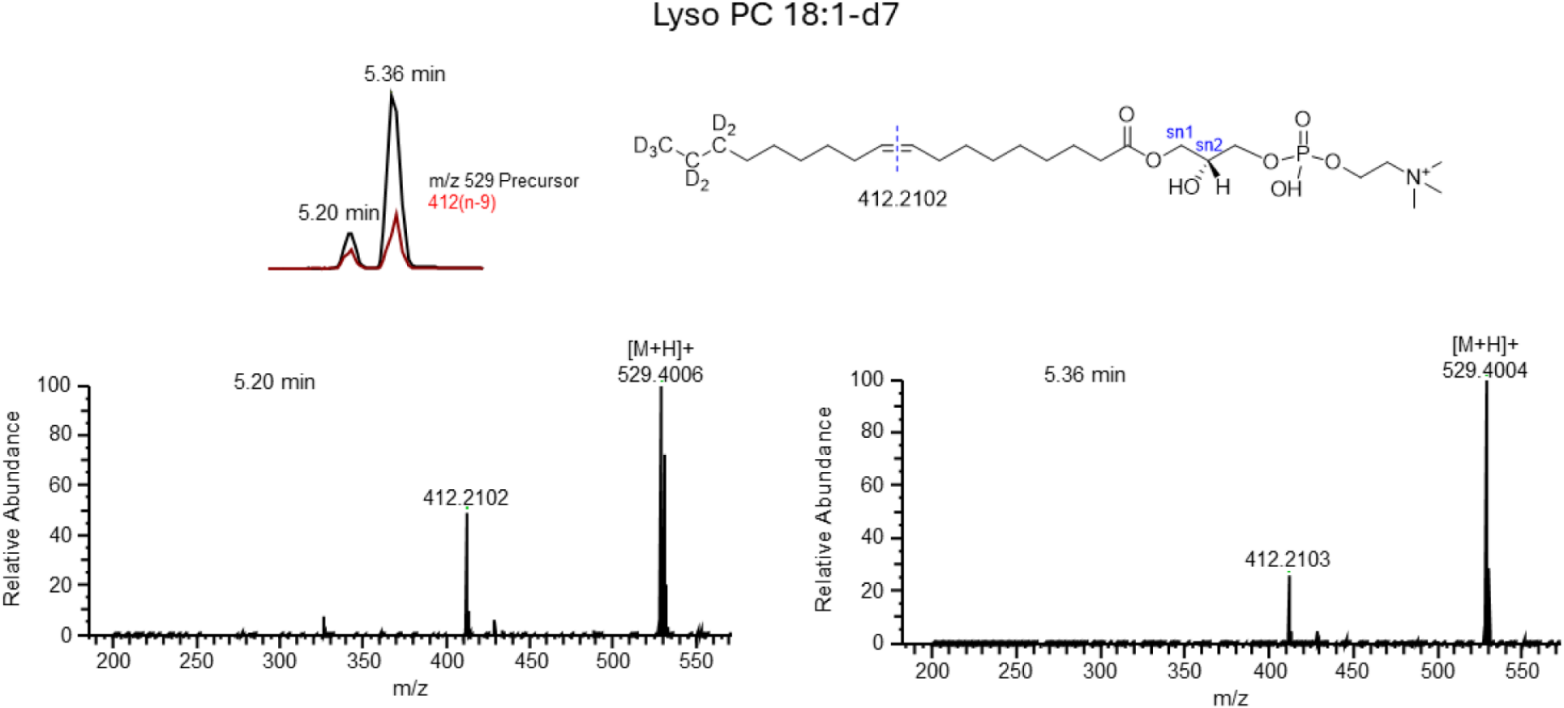
LC-OzESI-MS selected ion chromatograms and mass spectra of *sn* isomers of Lyso PC 18:1-d7.

### Application of LC-OzESI-MS to Identify Unsaturated PC Species in Mouse Liver Lipid Extract

Finally, we applied LC-OzESI-MS to a complex lipid extract from biological samples. It is noted that LC-OzESI-MS alone cannot identify unsaturated lipids, as the MS1 scan alone cannot provide the necessary head and tail information needed for lipid structural elucidation. A two-step approach was utilized instead, with the first step using LC-MS/MS with data-dependent scanning and ozone off to identify the putatively annotated lipids, then applying LC-OzESI-MS in the following ozone-on runs to assign the C=C position in a targeted fashion, relying on the accurate mass and retention time from the ozone-off runs to interrogate the MS1 spectrum under ozone on. For PC species, we found that most of the time this strategy works well in that, for the 46 unsaturated PC species identified from the LC-MS/MS runs, 39 can be confidently annotated for their C=C position, but this critically depends on matrix effects, i.e., the coeluting lipid species. For example, PC 16:0_18:2 had a very clean MS1 spectrum at 12.61 min (Figure 8A), with the precursor ion, aldehyde, and Criegee ions corresponding to n-6 and n-9 positions on the FA 18:2, and the ozonide adduct ion all observed without any interferences. The situation gets a little complicated for PC 18:0_18:1, but it still works well, as the major isomer containing FA 18:1 (n-9) eluted at 16.17 min and had a very clean spectrum, the minor, early-eluting isomer, eluting at 16.02 min, composed of FA 18:1 (n-7), had a more complicated MS1 spectrum, where, in addition to the ions corresponding to the precursor and aldehyde fragment for PC 18:0_18:1 (n-7), a lipid species with *m*/*z* 794.7102 and an ion with *m*/*z* 684.5574 appeared, the gap of 110 Da indicates a monounsaturated C=C at the n-9 position (Figure 8B), which turned out to be an internal standard SM-*d*_9_18:1/22:1 (n-9). The situation gets more challenging for two coeluting species with very different abundance levels, as illustrated in Figure 8C. Here, PC 16:0_22:6 and PC 17:1_18:2 were coeluting at 11.98 min, but the former was much more abundant than the latter (the intensity of *m*/*z* 806.5699 is >200-fold higher than that of *m*/*z* 770.5682, as shown in the spectrum of Figure 8C). All C=Cs in the FA 22:6 can be confidently identified at n-3, -6, -9, -12, -15 and -18 positions, but the C=Cs in the FA 17:1 and 18:2 cannot, where no aldehyde/Criegee ions were observed except for *m*/*z* 660.4229, which corresponds to the aldehyde ion from breaking C=C at n-9 of FA 17:1. However, this ion overlaps with the n-12 C=C of FA 22:6 from PC 16:0_22:6. Its much higher intensity compared to the protonated precursor ion of PC 17:1_18:2 indicates that it cannot result exclusively from this precursor, therefore, none of the C=C positions in PC 17:1_18:2 can be identified.

**Figure 8:**
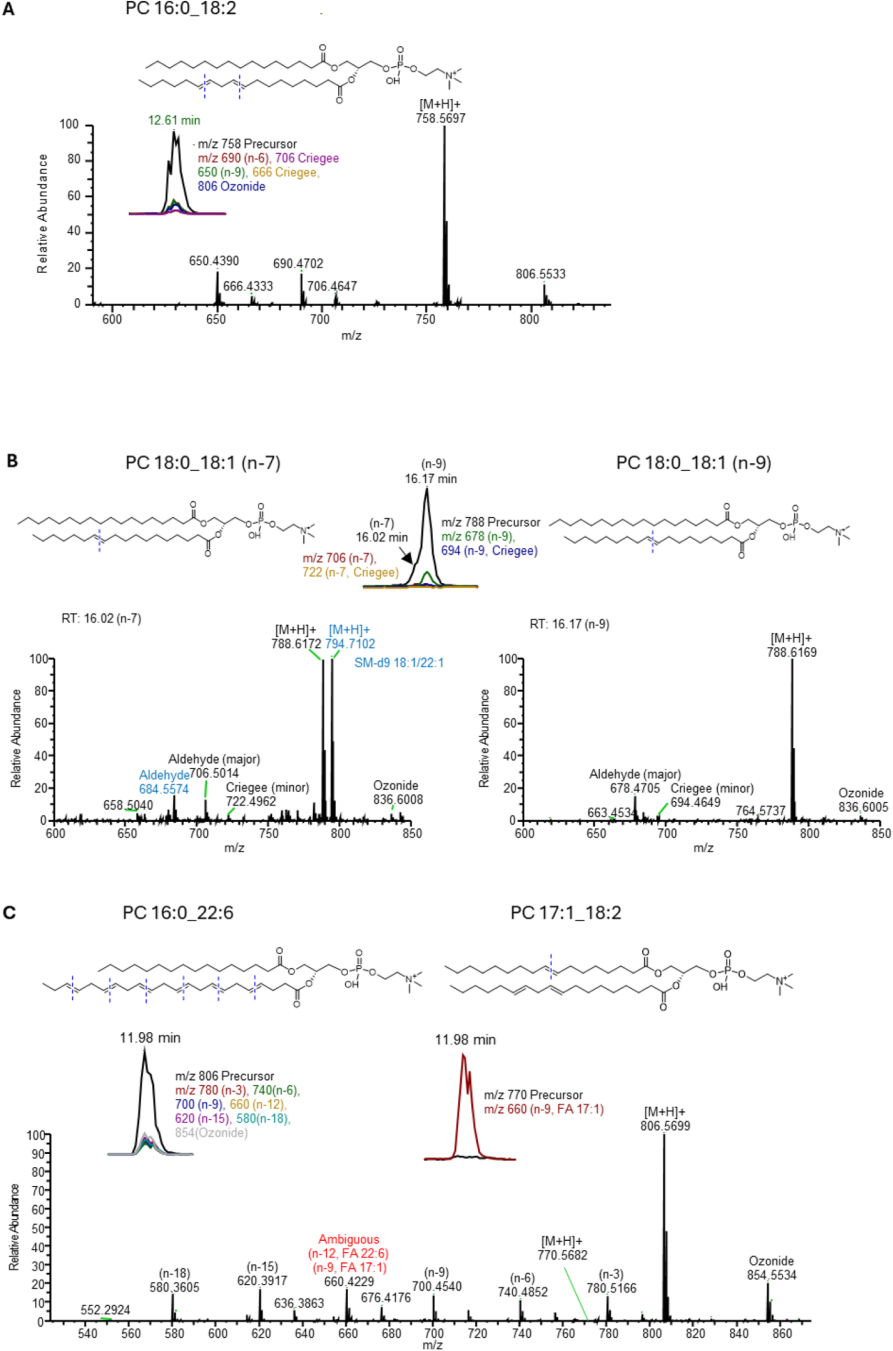
LC-OzESI-MS chromatogram and mass spectra of unsaturated PC species in lipid extracts from mouse liver. A) PC 16:0_18:2 without interference; B) Distinguishing isomeric PC 18:0_18:1 (n-7) and PC 18:0_18:1 (n-9), with the n-7 isomer having some interference from an internal standard lipid species SM-d9 18:1/22:1; C) Co-elution of PC 16:0_22:6 and PC 17:1_18:2, rendering the C=Cs in the low-abundance PC 17:1_18:2 unassigned.

## Conclusion

In this study, we developed an easily adoptable and broadly applicable OzESI setup by introducing ozone directly into the sheath-gas line of a standard HESI source on a Q Exactive HF mass spectrometer. This configuration allows efficient interaction of ozone with electrospray droplets without modifying the instrument hardware or compromising normal MS operation. The approach was validated across a wide range of lipid classes—including fatty acids, glycerophospholipids, sphingolipids, glycerolipids, and cholesteryl esters, as well as selected natural products such as cardol diene and coenzyme Q10. In all cases, diagnostic OzESI aldehyde product ions were reliably observed, enabling precise localization of double bonds and demonstrating the method’s generality. Reaction yields varied by lipid class and analyte, reflecting intrinsic chemical reactivity and potentially microdroplet effects, yet remained sufficient to allow unambiguous structural assignment. This sheath-gas-enrichment strategy provides a safe, robust, and easily adoptable approach for bond-specific ozonolysis in mass spectrometry. Furthermore, when coupling with LC separation, the coelution of C=C-specific aldehyde ions and the precursor facilitates the C=C assignment in complex lipid mixtures. Because of its high yield, although not illustrated in the current work, the generated ozonolysis products, including the aldehyde and ozonide ions can be further interrogated with traditional CID-MS/MS for further structural elucidation, as we previously demonstrated in elucidating the amino acid composition of a cyclic lipodepsipeptide, Orfamide N.^28^ Overall, our results establish this method as a versatile tool for structural elucidation of unsaturated lipids and natural products, extending the utility of LC-OzESI-MS to laboratories with standard instrumentation. We note that this approach works better with simple mixture samples that isomeric compounds can be separated chromatographically. For complex mixtures, such as lipid extracts from biological samples which may contain many isomeric lipids and C=C position-specific fragments, a two-stepped approach works moderately well, with the first step for LC-MS/MS-based unsaturated lipid identification under ozone-off conditions and the second step for LC-OzESI-MS with targeted interrogation of the MS1 spectrum, relying on the accurate mass and retention time of lipids identified in the ozone-off LC-MS/MS runs. We note that the overlapping of signals in mass spectra can arise in complex lipid extracts. In this case, OzID in ion mobility-enabled mass spectrometers^23^ or the OzNOxESI,^42^ a novel ozone-based ion chemistry recently reported by our lab, can fill in this gap, in which precursor ion selection followed by either ozonolysis or by collisional-induced dissociation tandem mass spectrometry, yielding characteristic product ions capable of unambiguously annotating C=C regioisomers.
